# Spinocerebellar Ataxia Type 2 Is Associated with the Extracellular Loss of Superoxide Dismutase but Not Catalase Activity

**DOI:** 10.3389/fneur.2017.00276

**Published:** 2017-06-13

**Authors:** Dennis Almaguer-Gotay, Luis E. Almaguer-Mederos, Raul Aguilera-Rodríguez, Roberto Rodríguez-Labrada, Dany Cuello-Almarales, Annelié Estupiñán-Domínguez, Luis C. Velázquez-Pérez, Yanetza González-Zaldívar, Yaimé Vázquez-Mojena

**Affiliations:** ^1^Center for the Research and Rehabilitation of Hereditary Ataxias (CIRAH), Holguín, Cuba

**Keywords:** CAG repeat, catalase, clinical phenotype, superoxide dismutase, spinocerebellar ataxia type 2

## Abstract

**Background:**

Spinocerebellar ataxia type 2 (SCA2) is an inherited and still incurable neurodegenerative disorder. Evidence suggests that pro-oxidant agents as well as factors involved in antioxidant cellular defenses are part of SCA2 physiopathology.

**Aim:**

To assess the influence of superoxide dismutase (SOD3) and catalase (CAT) enzymatic activities on the SCA2 syndrome.

**Method:**

Clinical, molecular, and electrophysiological variables, as well as SOD3 and CAT enzymatic activities were evaluated in 97 SCA2 patients and in 64 age- and sex-matched control individuals.

**Results:**

Spinocerebellar ataxia type 2 patients had significantly lower SOD3 enzymatic activity than the control group. However, there were no differences between patients and controls for CAT enzymatic activity. The effect size for the loss of patients’ SOD3 enzymatic activity was 0.342, corresponding to a moderate effect. SOD3 and CAT enzymatic activities were not associated with the CAG repeat number at the *ATXN2* gene. SOD3 and CAT enzymatic activities did not show significant associations with the age at onset, severity score, or the studied electrophysiological markers.

**Conclusion:**

There is a reduced SOD3 enzymatic activity in SCA2 patients with no repercussion on the clinical phenotype.

## Introduction

Spinocerebellar ataxia type 2 (SCA2) is an inherited neurodegenerative disorder reaching the highest worldwide prevalence rate in Holguín province, Cuba (1). SCA2 is caused by a CAG repeat expansion mutation on the first exon of the *ATXN2* gene. The *ATXN2* gene codes for ataxin-2. This is a ubiquitously expressed polyglutamine protein, which has been associated with RNA processing, translational regulation, endocytosis, and cell signaling (2, 3).

Little is known about the physiopathological mechanisms triggering SCA2 neurodegeneration. However, it has been suggested that oxidative stress might have an important role on the pathological processes (4, 5). Oxidative stress has been pointed out as a pathological mechanism for additional polyglutamine disorders. For instance, an increased production of reactive oxygen species (ROS) preceding cell death was revealed in a cell model for Huntington’s disease; ROS production was poly(Q) length-dependent (6). Similarly, in an inducible cell model for spinocerebellar ataxia type 7, the expression of mutated *ATXN7* gene produced a concomitant increase in ROS levels, aggregation of the disease protein, and cellular toxicity (7).

Additionally, decreased superoxide dismutase (SOD) and catalase (CAT) enzymatic activities have been probed in several models for polyglutamine disorders (7, 8). Besides, neuroprotective effects have been noticed for the SOD3 secreted by mesenchymal stem cells on cerebellar cells *in vitro* (9), and higher levels of CAT have been observed in a transgenic mouse model (YG8) for Friedreich’s ataxia (FRDA) (10), highlighting the significance of these enzymes in counteracting neurodegenerative processes.

SOD and CAT enzymes are core components of the body’s enzymatic antioxidant defenses to control ROS production. SOD and CAT enzymes, catalyzing two consecutive reactions, work to transform the superoxide free radical (^⋅^O^−^) to water. First, ^⋅^O^−^ is converted to hydrogen peroxide (H_2_O_2_) by SOD, while in a second step, H_2_O_2_ is converted into oxygen and water by CAT (11). SOD’s enzyme family is integrated by three members: SOD1 (cytoplasmic, Cu/Zn dependent: OMIM-147450), SOD2 (mitochondrial, Mn dependent: OMIM-147460), and SOD3 (extracellular, Cu/Zn dependent: OMIM-185490). Several mutations in SOD1 gene cause amyotrophic lateral sclerosis, probably by altering SOD1 enzymatic activity through the gain of aberrant functions like enhancing catalysis of tyrosine nitration by peroxynitrite (12).

Preliminary evidences have been obtained supporting a role for these enzymes in SCA2 physiopathology (4, 13). However, a deeper characterization of the influence of SOD and CAT enzymatic activities on the SCA2 clinical phenotype is needed to assess the potential value of these enzymes as disease biomarkers. Here, a study was conducted to assess the association of SOD3 and CAT enzymatic activities on the SCA2 clinical phenotype taking advantage of the largest SCA2 population worldwide.

## Materials and Methods

### Subjects and Study Design

A total of 161 subjects, 97 SCA2 patients and 64 healthy control individuals, were included in the study. A case–control design was applied to assess the association between SOD3 or CAT enzymatic activities and SCA2. Both affected and control subjects were sex and age matched; a maximal age difference of 2 years was allowed. The study protocol was approved by the Research Ethics Committee at the Center for Research and Rehabilitation of Hereditary Ataxias, and a written informed consent was obtained from the affected and control individuals included in the study.

### Clinical Evaluation

Spinocerebellar ataxia type 2 clinical diagnosis was based on the identification of gait ataxia, dysarthria, dysmetria, dysdiadochokinesia, and slowing of saccade eye movements. Age at onset (AO) was defined as the onset of motor impairment. Evolution time (ET) was defined as the time elapsed between clinical debut and the time when the last neurologic evaluation was made. Clinical severity was estimated by using the Scale for the assessment and rating of ataxia (SARA) (14).

### Assessment of Ocular Saccade Movements

Horizontal and vertical saccade eye movements were recorded binocularly with silver–silver chloride electrodes over right and left outer canthus and a two-channel Otoscreen AC electronystagmography (Jaeger-Toennies, Höchberg, Germany) with a band-pass filter of 0.02–70 Hz, a sensitivity of 200 μV/division, a time base of 1,000 ms/division, a time constant of 8 s, and a sampling rate of 200 Hz. Saccade eye movements were elicited with a circular white target subtending an angle of 0.7° on a black background. The distance between patient and monitor as well as the head position was controlled by chin/head supports. At least 10 horizontal centrifugal saccades in either direction were recorded for each 10°, 20°, 30°, and 60° predictable amplitudes. Comparison of independent calibrations at a 30° angle before and after all recordings was used to control against artifacts.

### Assessment of SOD3 and CAT Enzymatic Activities

Serum SOD3 and CAT enzymatic activities—expressed as U/L and UI/L, respectively—were measured at 37°C by using standard methods (15, 16). Buffer EDTA (5 mM)-Tris–HCl (1 M) (pH = 8), as well as pyrogallol and H_2_O_2_ substrates were used. One unit of SOD3 enzymatic activity was defined as the amount of SOD3 that inhibits the pyrogallol auto-oxidation to a 50%. Similarly, one unit of CAT enzymatic activity was defined as the amount of CAT that catalyzes the reduction of 1 µmol of hydrogen peroxide per minute.

Serum samples were obtained from blood by centrifugation at 3,000 rpm and 4°C during 10 minutes. Samples were freezed at −20°C until used. Every sample was analyzed in triplicate using a Biomate-3 Spectrophotometer (Thermo Spectronic Company, USA).

### CAG Repeat Measurement and Molecular Testing for SCA2

Genomic DNA was obtained from peripheral blood using a modified *Nucleon II* standard protocol (ScotLab, Scotland). The CAG repeat at the *ATXN2* locus was amplified by PCR using UH10/UH13 oligonucleotide primers (17). The CAG repeat number was measured by polyacrylamide gel electrophoresis in an ALFexpress II DNA analyzer (Amersham Biosciences, Sweden), with the use of internal and external standard size markers and the Allele Links software (1.0) for processing. Individuals with 32 or more CAG repeats were considered as mutation carriers (18).

### Statistical Analysis

A power analysis was conducted to estimate the minimum sample size needed to achieve a 90% statistical power. It was obtained that a sample size of 70 patients and 70 controls was needed to achieve a 90% statistical power for an alpha of 0.05 and a medium effect size.

Descriptive statistics were used to assess central tendencies and dispersion of data. Kolmogorov–Smirnov test was used to assess the normality of data distribution. In case of data not fulfilling the normality assumption, mathematic transformation of the variables was applied.

Student’s *t*-test was used to compare SOD3 and CAT enzymatic activities between patients and controls and to assess the effect of sex on enzymatic activities. Pearson’s correlation test was used to assess the associations between SOD3 and CAT enzymatic activities and between clinical variables and SOD3 and CAT enzymatic activities. Multiple lineal regression analyses were conducted to assess the predictive values of SOD3 and CAT enzymatic activities on the AO, SARA score, maximal saccade velocity, and saccade’s latency. Statistical significance was defined as *p* < 0.05. Statistic 6.0 software was used for data processing.

## Results

### Seric SOD3 and CAT Enzymatic Activities in the Studied Sample

Seric SOD3 and CAT enzymatic activities were measured in 161 individuals—66 females and 95 males—aged 15–75 years (mean age: 40.7 years). Neither SOD3 nor CAT enzymatic activities showed a Gaussian distribution (*p* < 0.0001).

SOD3 enzymatic activity varied between 1.16 and 4.78 KU/L, while CAT enzymatic activity was in the range of 3.52–67.61 KUI/L. Mean (SD) SOD3 enzymatic and CAT enzymatic activities were 2.41 (0.80) KU/L and 20.65 (10.85) KUI/L, respectively. There were no significant differences for LogSOD (*t* = −0.010, *p* = 0.99) or LogCAT (*t* = −1.080, *p* = 0.28) activities between males and females. In addition, no significant correlations were found between age and LogSOD3 (*p* = 0.366) or LogCAT (*p* = 0.275) activities. Log transformed SOD3 and CAT enzymatic activities were highly significantly correlated (*r* = 0.218, *p* = 0.005).

### SOD3’s and CAT’s Activities in SCA2 Patients and Controls

Seric SOD3 and CAT enzymatic activities were determined in 64 SCA2 individuals and in 64 age- and sex-matched healthy controls. Among patients, the mean (SD) SOD3 and CAT enzymatic activities were 2.14 (1.45) KU/L and 18.62 (1.48) KUI/L, respectively. On the contrary, among control individuals, the mean (SD) SOD3 and CAT enzymatic activities were 2.40 (1.35) KU/L and 17.78 (1.78) KUI/L, respectively.

**Figure 1:**
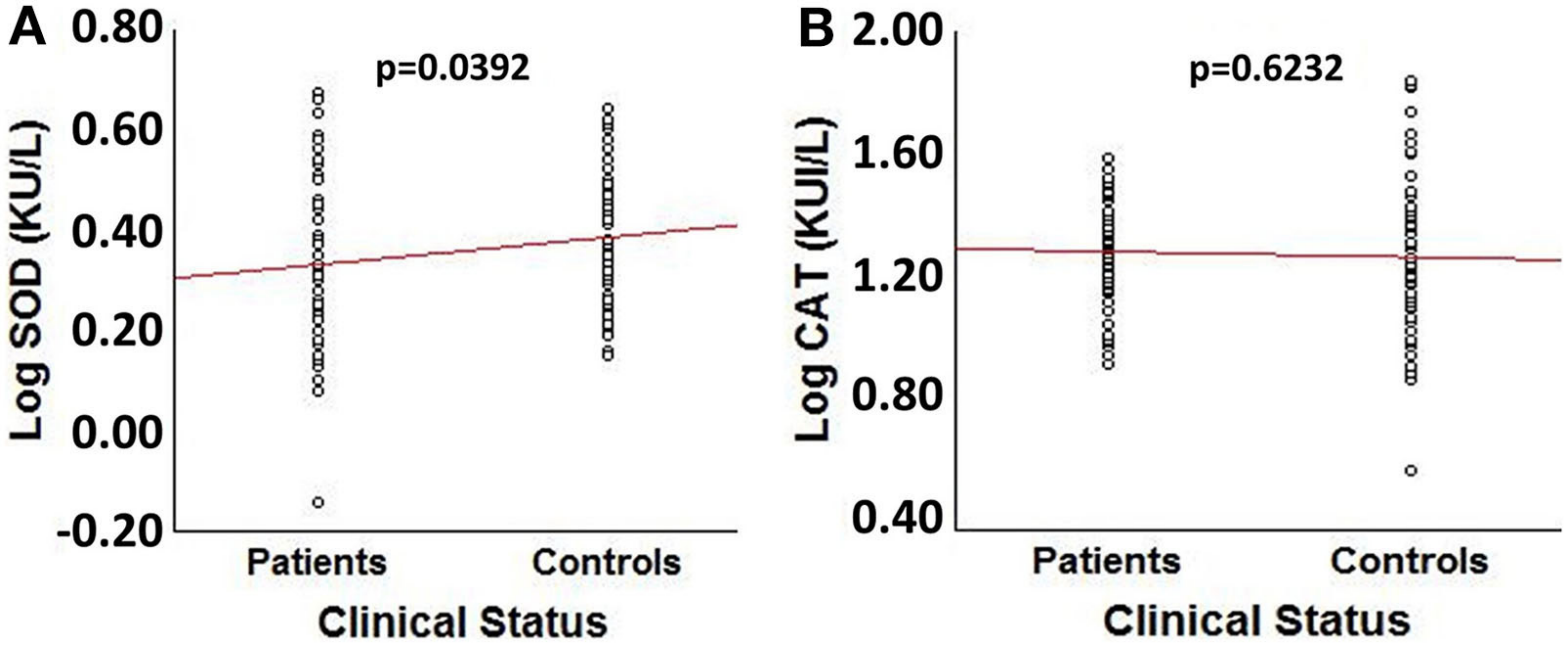
Comparison of log transformed SOD3 and catalase (CAT) enzymatic activities between spinocerebellar ataxia type 2 patients (*n* = 64) and sex- and age-matched control individuals (*n* = 64). **(A)** Box plot for LogSOD3 activities. **(B)** Box plot for LogCAT activities.

Comparison of log transformed SOD3 and CAT enzymatic activities between patients and controls showed a significant decrease in patients’ SOD3 activity (*t* = −2.084, *p* = 0.039) (Figure 1A). However, no significant differences were obtained for CAT activity or between patients and controls (*p* = 0.620) (Figure 1B). The effect size for SOD3 enzymatic activity was 0.342, corresponding to a moderate effect; meanwhile, the effect size for CAT’s activity was 0.093, corresponding to a very small effect.

No significant differences were obtained for Log 1/(SOD3/CAT) quotient between patients and controls (*p* = 0.070). Log transformed SOD3 and CAT enzymatic activities were not significantly influenced by sex in SCA2 patients (male/female: SOD3 (KU/L) = 0.33/0.33, *p* = 0.964; CAT (KUI/L) = 1.28/1.25, *p* = 0.555). A similar picture was obtained in the control group (male/female: SOD3 (KU/L) = 0.39/0.38, *p* = 0.945; CAT (KUI/L) = 1.24/1.27, *p* = 0.686). No significant correlations were obtained between age and SOD3 or CAT enzymatic activities (*p* > 0.05).

### Association between SOD3 and CAT Enzymatic Activities and Disease Severity and Progression

Descriptive statistics for clinical and molecular markers in the study are shown in Table 1. Patient’s age did not show significant correlations with log transformed SOD3 (*r* = −0.0333, *p* = 0.7462) or CAT (*r* = 0.1825, *p* = 0.0736) enzymatic activities, or the Log 1/(SOD3/CAT) quotient (*r* = 0.0788, *p* = 0.450).

**Table 1:** Descriptive statistics for clinical and molecular markers under study.

Variables	*N*	Mean	Range	SD	SEM
Age (years)	97	42.6	19–75	11.3	1.1
*ATXN2*_exp_ (CAG repeats)	97	39.9	32–52	3.4	0.3
Age at onset (AO) (years)	97	30.6	11–62	11.3	1.2
Evolution time (years)	97	12.0	1–33	6.5	0.7
SOD3 activity (KU/L)	97	2.3	1.2–4.8	0.8	0.1
Catalase (CAT) activity (KUI/L)	97	20.4	7.3–56.3	8.3	0.8
SOD3/CAT	97	0.13	0.04–0.50	0.07	0.007
**Maximal saccade velocity (°/s)**					
10°	41	185.7	15.2–778.0	129.9	20.0
30°	41	291.2	23.9–1,026.0	183.3	28.6
60°	41	324.5	36.3–1,193.0	208.5	32.6
**Saccade latency (ms)**					
10°	43	223.5	115.0–518.0	83.2	12.8
30°	43	248.4	107.0–616.4	89.0	13.6
60°	43	275.5	127.0–621.4	95.8	14.6
Scale for the assessment and rating of ataxia (SARA) score (units)	46	16.7	10.0–25.0	4.1	0.6
SARA/TE (units/year)	46	1.79	0.56–4.0	0.90	0.1

As expected, a significant correlation between the AO and the CAG repeat number in *ATXN2* expanded alleles was obtained. However, the CAG repeat number did not show any significant correlation with the log transformed SOD3 (*r* = 0.1518, *p* = 0.1377) or CAT (*r* = −0.1577, *p* = 0.1229) enzymatic activities, neither with the log transformed 1/(SOD3/CAT) quotient (*r* = −0.1812, *p* = 0.081). Also, there were no significant correlations between the AO and the log transformed SOD3 (*r* = −0.0225, *p* = 0.8265) or CAT (*r* = 0.1382, *p* = 0.1770) enzymatic activities, neither with the log transformed 1/(SOD3/CAT) quotient (*r* = 0.0581, *p* = 0.578). The no significant associations between the AO and SOD3 and CAT enzymatic activities were verified by multiple linear regression adjusting for *ATXN2* CAG repeat number (*p* > 0.05). In addition, the ET was not significantly correlated with any of the measured enzymatic activities (logSOD3: *r* = −0.0179, *p* = 0.861; logCAT: *r* = 0.0932, *p* = 0.361).

Since slowing saccade ocular movements is a distinctive clinical sign in SCA2, potential associations with SOD3 and CAT enzymatic activities were investigated. Maximal saccade velocity at 10°, 30°, and 60° were significantly correlated with the CAG repeat number. On the contrary, saccade latencies were not significantly correlated with the CAG repeat number (*p* > 0.05). In addition, the ET, log transformed SOD3 and CAT enzymatic activities, and the 1/(SOD3/CAT) quotient, were not significantly correlated with the maximal saccade velocity neither with saccade latency (Table 2). The lack of significant correlations between the maximal saccade velocity or the saccade latency and log transformed SOD3 and CAT enzymatic activities, and the 1/(SOD3/CAT) quotient, were verified by multiple linear regression adjusting for age and *ATXN2* CAG repeat number (*p* > 0.05).

**Table 2:** Molecular and physiological factors modulating the spinocerebellar ataxia type 2 patients’ saccade eye movements.

Independent variables	Saccade movement variables (Pearson correlation coefficient/*p*-value)					
	Log velocity (°/s)			Log latency (ms)		
	10°	30°	60°	10°	30°	60°
Age (years)	0.54/0.001**	0.39/0.015*	0.28/0.080	−0.29/0.071	−0.25/0.115	−0.17/0.321
*ATXN2* expanded alleles (CAG repeats)	−0.49/0.003**	−0.38/0.018*	−0.33/0.039*	0.23/0.152	0.29/0.067	0.24/0.471
Evolution time (years)	0.11/0.534	−0.06/0.703	−0.18/0.274	0.02/0.920	0.03/0.875	0.05/0.755
Log SOD3 activity (KU/L)	0.04/0.812	0.004/0.980	0.006/0.974	−0.11/0.492	−0.08/0.629	−0.30/0.077
Log CAT activity (KUI/L)	−0.01/0.942	−0.02/0.918	−0.04/0.809	−0.02/0.907	0.11/0.489	0.05/0.772
Log 1/(SOD3/CAT)	−0.04/0.805	−0.02/0.901	−0.04/0.787	0.07/0.681	0.17/0.286	0.30/0.079

*Significant association; **Highly significant association.

In a sample of 46 SCA2 patients for which information about SARA score was available, a significant correlation between SARA score and ET (*r* = 0.4803, *p* = 0.001), and *ATXN2* CAG repeat number (*r* = 0.2985, *p* = 0.044) was found. The *ATXN2* CAG repeat number was also significantly correlated with the disease’s progression rate (SARA/Log ET) (*r* = 0.3962, *p* = 0.006).

However, neither the SARA score nor the progression rate were significantly correlated with the log transformed SOD3 (*r* = 0.0878, *p* = 0.562; *r* = 0.2358, *p* = 0.115, respectively), and CAT (*r* = 0.0296, *p* = 0.845; *r* = 0.0270, *p* = 0.859, respectively) enzymatic activities, neither with the 1/(SOD3/CAT) quotient (*r* = −0.152, *p* = 0.319; *r* = −0.079, *p* = 0.605, respectively). By multiple linear regression, the lack of significant associations between these clinical variables and the log transformed SOD3 and CAT enzymatic activities and the 1/(SOD3/CAT) quotient was verified, once adjusted for the ET and the *ATXN2* CAG repeat number (*p* > 0.05).

## Discussion

### SOD3 and CAT Enzymatic Activities in SCA2 Patients

By measuring biologic markers of oxidative damage, an important role for oxidative stress as part of the molecular physiopathology of neurodegenerative disorders like Alzheimer’s and Huntington’s diseases and FRDA (19–21) has been suggested. Additionally, in patients and cell models for spinocerebellar ataxia type 3, it has been shown that oxidative stress could be associated with a significant alteration of SOD and CAT enzymatic activities (22, 23). Preliminary results also pointed to a role for oxidative stress as part of the SCA2 physiopathological processes, although contradictory results have been obtained regarding SOD3 and CAT enzymatic activities, probably due to limited sample sizes (4, 13). More recently, an increase in SOD2 and CAT mRNA and protein levels in fibroblasts of SCA2 patients relative to controls was found, highlighting the significance of these enzymes in counteracting SCA2 neurodegenerative processes (24).

In this study, SCA2 patients showed a moderate decrease in SOD3 activity relative to control individuals, but no difference for CAT activity was noted, suggesting that mild accumulation of superoxide free radical could be taking place in SCA2. The lack of association between SCA2 and CAT enzymatic activity could be due to the lack of statistical power. Actually, based on *a posteriori* effect sizes calculations, a much larger sample will be needed to detect any significant effects of CAT enzymatic activity on SCA2.

SOD3 activities in the affected patients were not associated with the CAG repeat number at *ATXN2* expanded alleles, suggesting that patients’ SOD3 activity loss is not directly associated with the primary physiopathological events. This result partially agrees with a previous study conducted in SCA2 patients (4), and similar results have been obtained in cellular and mice models for Huntington’s disease (8, 25).

A possible implication of a decreased SOD3 enzymatic activity in SCA2 patients is an increased susceptibility to oxidative stress. It has been showed that SOD3 null mutant mice develop normally and remain healthy until at least 14 months of age; however, when stress is produced by exposure to >99% oxygen, mutant mice display a considerable reduction in survival time compared to wild-type mice, and an earlier onset of severe lung edema (26). These findings suggest that while under normal physiological conditions additional antioxidant systems may compensate for the loss of SOD3, the exposure to stress make these systems unable to provide adequate protection. Similar events could be occurring in SCA2 patients who are under the prolonged stressing and neurotoxic effects of expanded ataxin-2 (27), then favoring the occurrence of oxidative stress and the aggravation of the clinical phenotype.

### Clinical Implications of the SOD3 Enzymatic Activity Loss

In polyglutamine diseases, the CAG repeat expansion mutation is the main genetic factor influencing on clinical variability (28). In this study, a significant influence of the *ATXN2* CAG repeat number on several clinical markers was showed, explaining about 45% of the age at disease onset, 11% of maximal saccade velocity at 60°, 9% of SARA score, and 16% of the progression rate variability, suggesting the existence of additional factors, genetic and environmental factors, acting as modifiers of the clinical phenotype.

Oxidative stress has been proposed as a potentially important component of SCA2 physiopathology, leading to the aggravation of the clinical phenotype (4, 5). Preliminary evidence point to an association between the peroxidation potential and the saccade latency, and between the ferric reducing ability of plasma and the peroneal nerve latency. However, no significant association has been found for SOD3 enzymatic activity and neurophysiological parameters (4). Similarly, in the present study, no significant association was noticed between SOD3 or CAT enzymatic activities and any of the studied clinical markers (AO, maximal saccade velocity, saccade latency, SARA score, and disease progression rate). In line with these results, it has been shown in a yeast model for Huntington’s disease that overexpression of superoxide dismutases, CATs, and glutathione reductases did not consistently confer protection against Htt103Q-mediated toxicity. Also, no reduction in mutant htt-induced caspase activation was noticed in PC12 cells overexpressing CAT or superoxide dismutase 1, even when there was an increase in both SOD and CAT enzymatic activities (29).

Based on the results from this study, it could be suggested that neither SOD3 nor CAT enzymatic activities are potentially useful as biomarkers of SCA2 severity and progression. However, since both SOD3 and CAT enzymatic activities were only measured at the systemic level, further studies will be needed in additional levels—i.e., cerebrospinal fluid—to verify the results and to clarify the role of SOD3 and CAT enzymatic activities in the SCA2 physiopathology. In addition, even when a very large cohort of SCA2 patients was studied, extending this study to presymptomatic individuals will be adding new relevant information regarding the role of these enzymes in early stages of the disease process.

## Conclusion

There is a reduced SOD3 enzymatic activity in SCA2 patients with no obvious impact on the clinical phenotype.

## Ethics Statement

The study protocol was approved by the Research Ethics Committee at the Center for Research and Rehabilitation of Hereditary Ataxias, and a written informed consent was obtained from the affected and control individuals.

## Author Contributions

DA-G, LA-M, and RA-R contributed to the conception and design of the work. DA-G, LA-M, RA-R, RR-L, DC-A, AE-D, LV-P, YG-Z, and YV-M contributed to the acquisition, analysis, and interpretation of data for the work. DA-G and LA-M wrote the paper. DA-G, LA-M, YG-Z, and YV-M revised the paper critically. DA-G, LA-M, RA-R, RR-L, DC-A, AE-D, LV-P, YG-Z, and YV-M approved the final version of the paper to be published.

## Conflict of Interest Statement

The authors declare that the research was conducted in the absence of any commercial or financial relationships that could be construed as a potential conflict of interest.
